# Adaptive Thermogenesis in a Mouse Model Lacking Selenoprotein Biosynthesis in Brown Adipocytes

**DOI:** 10.3390/ijms22020611

**Published:** 2021-01-09

**Authors:** Lucia A. Seale, Ashley N. Ogawa-Wong, Ligia M. Watanabe, Vedbar S. Khadka, Mark Menor, Daniel J. Torres, Bradley A. Carlson, Dolph L. Hatfield, Marla J. Berry

**Affiliations:** 1 Department of Cell and Molecular Biology, John A. Burns School of Medicine, University of Hawaii at Manoa, Honolulu, HI 96813, USA; anogawa@hawaii.edu (A.N.O.-W.); ligia_watanabe@usp.br (L.M.W.); djtorr@hawaii.edu (D.J.T.); 2 Pacific Biomedical Research Center, School of Ocean and Earth Science and Technology, University of Hawaii at Manoa, Honolulu, HI 96822, USA; mberry@hawaii.edu; 3 Department of Quantitative Health Sciences, John A. Burns School of Medicine, University of Hawaii at Manoa, Honolulu, HI 96822, USA; vedbar@hawaii.edu (V.S.K.); mmenor@hawaii.edu (M.M.); 4 Molecular Biology of Selenium Section, Mouse Genetics Program, National Cancer Institute, National Institutes of Health, Bethesda, MD 20892, USA; carlsonb@dc37a.nci.nih.gov (B.A.C.); hatfielddolph@gmail.com (D.L.H.)

**Keywords:** Sec-tRNA^[Ser]Sec^, selenoproteins, brown adipose tissue

## Abstract

Selenoproteins are a class of proteins with the selenium-containing amino acid selenocysteine (Sec) in their primary structure. Sec is incorporated into selenoproteins via recoding of the stop codon UGA, with specific *cis* and *trans* factors required during translation to avoid UGA recognition as a stop codon, including a Sec-specific tRNA, tRNA^[Ser]Sec^, encoded in mice by the gene *Trsp*. Whole-body deletion of *Trsp* in mouse is embryonically lethal, while targeted deletion of *Trsp* in mice has been used to understand the role of selenoproteins in the health and physiology of various tissues. We developed a mouse model with the targeted deletion of *Trsp* in brown adipocytes (*Trsp^f/f^-Ucp1-Cre*^+/−^), a cell type predominant in brown adipose tissue (BAT) controlling energy expenditure via activation of adaptive thermogenesis, mostly using uncoupling protein 1 (Ucp1). At room temperature, *Trsp^f/f^-Ucp1-Cre*^+/−^ mice maintain oxygen consumption and Ucp1 expression, with male *Trsp^f/f^-Ucp1-Cre*^+/−^ mice accumulating more triglycerides in BAT than both female *Trsp^f/f^-Ucp1-Cre*^+/−^ mice or *Trsp^f/f^* controls. Acute cold exposure neither reduced core body temperature nor changed the expression of selenoprotein iodothyronine deiodinase type II (Dio2), a marker of adaptive thermogenesis, in *Trsp^f/f^-Ucp1-Cre^+/−^* mice. Microarray analysis of BAT from *Trsp^f/f^-Ucp1-Cre^+/−^* mice revealed glutathione S-transferase alpha 3 (*Gsta3*) and ELMO domain containing 2 (*Elmod2*) as the transcripts most affected by the loss of *Trsp*. Male *Trsp^f/f^-Ucp1-Cre^+/−^* mice showed mild hypothyroidism while downregulating thyroid hormone-responsive genes *Thrsp* and *Tshr* in their BATs. In summary, modest changes in the BAT of *Trsp^f/f^-Ucp1-Cre ^+/−^* mice implicate a mild thyroid hormone dysfunction in brown adipocytes.

## 1. Introduction

Selenoproteins are a small group of proteins containing the micronutrient selenium in their molecule. These proteins are involved in redox reactions that can reduce oxidative stress, activate thyroid hormones, and act on endoplasmic reticulum-associated degradation of misfolded proteins. There are only 24 and 25 selenoproteins in mice and humans, respectively. Nevertheless, selenoproteins exert a profound effect in several aspects of health, including the pathophysiology of different types of cancer [1,2,3], autoimmune diseases [4], irritable bowel syndrome [5], type 2 diabetes [6,7], and thyroid disorders [8,9].

A hallmark feature of selenoproteins is the incorporation of selenium as the amino acid selenocysteine (Sec) in their primary structure. Sec is encoded by the UGA codon, also recognized as a stop codon. Circumvention of the stop recognition occurs due to a combination of *cis* and *trans* factors that have been extensively reviewed elsewhere [10,11,12,13,14,15,16,17,18]. Notably, one of the key factors is the specific tRNA^[Ser]Sec^, required for successful insertion of Sec during translation of selenoproteins, and encoded by the gene *Trsp*.

Total loss of *Trsp* in mice is embryonically lethal [19], a finding that highlights how essential selenoproteins are for life. To demonstrate the role of selenoproteins in various organs, tissues and cell types, targeted deletion of *Trsp* in specific cell types has been carried out for decades, with surprising consequences [10,20,21]. However, the targeted deletion of *Trsp* in brown adipocytes, a cell type critical for thermoregulation and energy expenditure in mammals, had not been attempted.

Brown adipocytes are the most abundant cell type found in the brown adipose tissue (BAT), the main site for adaptive thermogenesis in rodents, and subpopulations of brown adipocytes present distinct thermogenic potential [22,23]. Mammalian adaptive thermogenesis is classically activated upon exposure to cold or after caloric overload. BAT cold-induced adaptive thermogenesis relies primarily on the actions of uncoupling protein 1 (Ucp1), a proton pump localized in the inner membrane of mitochondria of brown adipocytes that dissipates energy from ATP synthesis as heat [24,25]. Ucp1 expression is synergistically upregulated after activation of beta-adrenergic signaling and by thyroid hormone 3,3′,5-triiodothyronine (T3) [26]. Interestingly, T3 is mostly produced locally in cells, after the removal of one iodine from thyroxine (T4), the main prohormone from the thyroid gland. Conversion of T4 into active T3 in brown adipocytes is catalyzed by iodothyronine deiodinase type 2 (Dio2). Dio2 is a selenoprotein highly expressed in activated brown adipocytes and essential for cold-induced thermogenesis [27], allowing for the increased Ucp1 expression [28,29]. Mice with a disruption of Dio2 cannot properly carry out adaptive thermogenesis, either by cold exposure or caloric overload at room temperature [28,30,31]. Besides Dio2, glutathione peroxidases 1, 3, and 4 (Gpx1, Gpx3, and Gpx4, respectively), thioredoxin reductases 1, 2 and 3 (Txnrd1, Txnrd2, and Txnrd3, respectively), selenoproteins H, O, T and P (SelenoH, SelenoO, SelenoT, and SelenoP, respectively), and selenophosphate synthetase 2 (Sephs2) have also been found in BAT using a proteomics analysis [32], but the role of these selenoproteins in BAT function is unknown.

We report the generation of a mouse model with a targeted deletion of the gene for *Trsp* in brown adipocytes, the *Trsp^f/f^-Ucp1-Cre^+/−^* mice, to characterize the influence of selenoproteins in BAT function. These mice thermoregulate without changes in energy expenditure or Ucp1 expression, and surprisingly respond to acute cold exposure adequately. We also observed novel genes that respond to the lack of *Trsp* in brown adipocytes both at room temperature and after acute cold exposure, particularly those involved in methylation pathways and response to thyroid hormone levels.

## 2. Results

### 2.1. Generation of the Trsp^f/f^-Ucp1-Cre^+/−^ Mice

**Figure 1:**
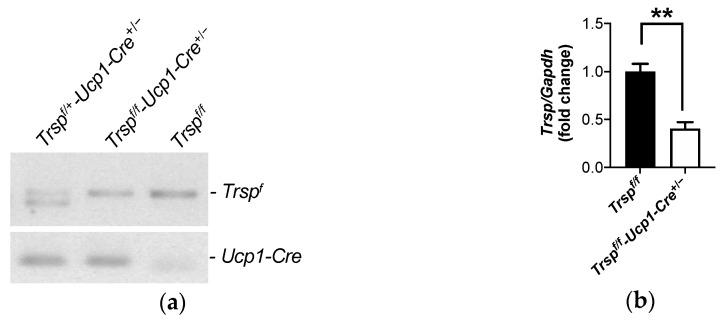
Genotype confirmation of *Trsp^f/f^-Ucp1-Cre^+/−^* after breeding. (**a**) PCR result of genotyping, showing the heterozygote mouse DNA with the doublet (1.1 and 0.9 kb) for *Trsp^f^* and *Trsp^+^* in the first lane, the *Trsp^f/f^-Ucp1-Cre^+/−^* mice in the second lane, and the *Trsp^f/f^* used as controls in the third lane. *Trsp^f/+^-Ucp1-Cre^+/−^* heterozygote mice were not used in experiments. **(b)** *Trsp* expression as measured by qPCR of whole brown adipose tissue (BAT). Values are means ± SD, *n* = 7. ** represents *p* < 0.01 after unpaired Student’s *t*-test.

We developed a transgenic mouse lacking the *Trsp* gene in brown adipocytes, *Trsp^f/f^-Ucp1-Cre^+/−^*. At room temperature, the *Ucp1-Cre* system has been previously shown to specifically express Cre recombinase only in brown adipocytes of the interscapular BAT [33], which assured specific *Trsp* deletion in this cell type. Deletion of the *Trsp* gene was confirmed by genotyping for both the floxed gene and for the presence of the *Ucp1-Cre* sequence (Figure 1a). BAT expression of the *Trsp* tRNA was reduced by ~60% (Figure 1b), with remaining *Trsp* expression possibly reflecting its presence in other cell types within the tissue.

### 2.2. Trsp^f/f^-Ucp1-Cre^+/−^ Mice Maintain Energy Expenditure

**Figure 2:**
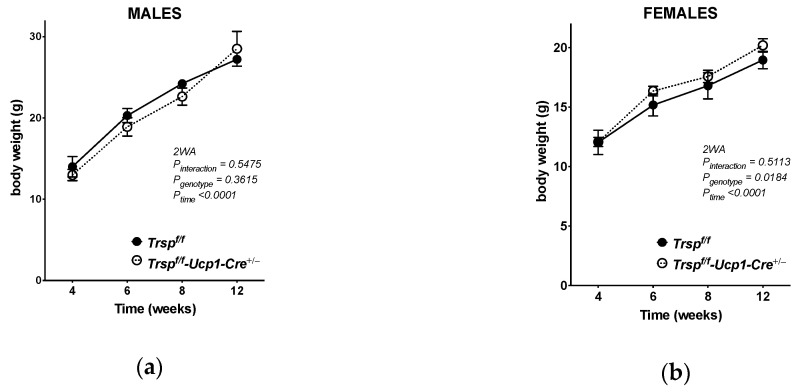

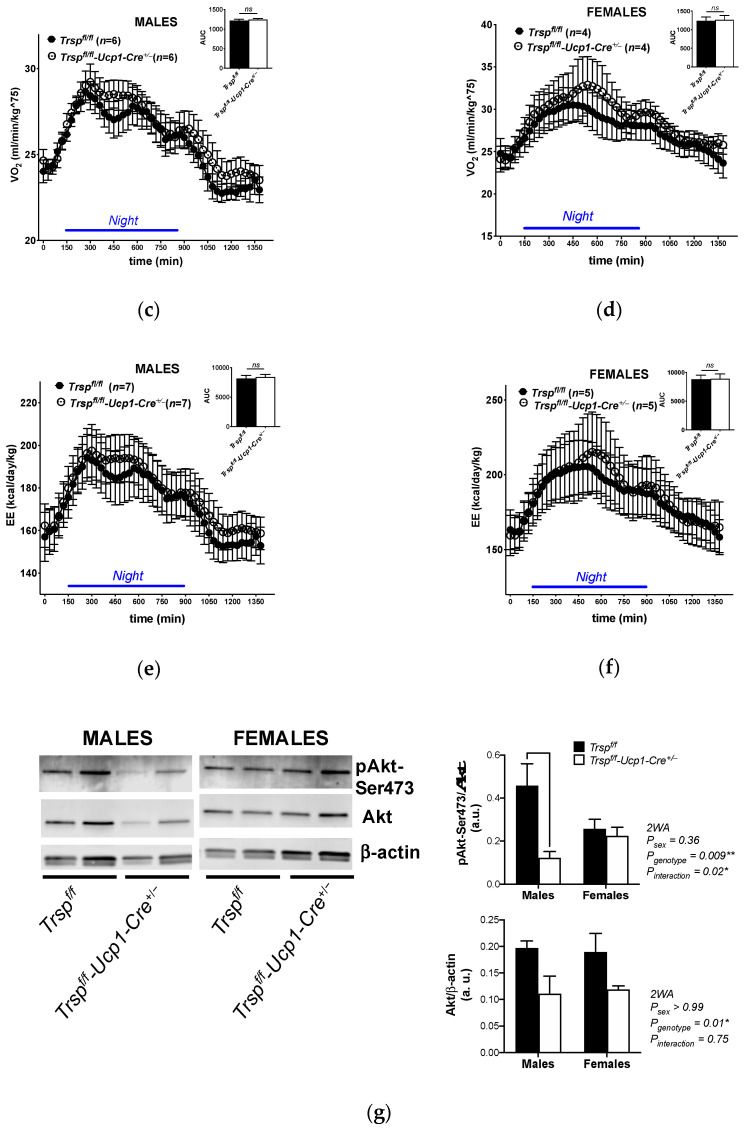
Metabolic overview of *Trsp^f/f^-Ucp1-Cre^+/−^* mice. Body weight assessment of male (**a**) and female (**b**) *Trsp^f/f^-Ucp1-Cre^+/−^* mice over 8 weeks after weaning. (**c**,**d**) VO_2_ consumption in male and female in the *Trsp^f/f^-Ucp1-Cre^+/−^* mice, respectively. (**e**,**f**) Energy expenditure calculations of male and female mice, respectively, considering VCO_2_ as well (VCO_2_ not shown). (**g**) Phosphorylated Akt at the Ser473 residue (pAkt-Ser473) and Akt levels in the gonadal WAT of male and female mice. Values are means ± SD. *p*-values were calculated in (**a**,**b**,**g**) after two-way ANOVA (2WA) followed by Bonferroni’s post-test, while for (**c**–**f**), *p*-values for the area under the curve (AUC) were calculated using Student’s *t*-test. The sample size is displayed in graphs, except in (**g**), where *n* = 6/sex. * *p* < 0.05, ** *p* < 0.01, and *ns*, non-significant. Black bars are *Trsp^f/f^* mice, and white bars are *Trsp^f/f^-Ucp1-Cre^+/−^* mice.

*Trsp^f/f^-Ucp1-Cre^+/−^* male mice showed no significant weight gain differences over time compared to *Trsp^f/f^* littermate control males (Figure 2a), while female *Trsp^f/f^-Ucp1-Cre^+/−^* were modestly heavier (Figure 2b) at 12 weeks of age compared to *Trsp^f/f^* littermate controls (*p* = 0.08). Brown adipocytes are the main site of adaptive thermogenesis in mice; hence, accounting for energy expenditure, we assessed the VO_2_ consumption and total energy expenditure of male and female *Trsp^f/f^-Ucp1-Cre^+/−^* mice, after placing mice in metabolic chambers for 24 h. We determined that neither VO_2_ consumption (Figure 2c,d) nor their total energy expenditure (Figure 2e,f) were altered in *Trsp^f/f^-Ucp1-Cre^+/-^* mice at room temperature, as calculated by the average of the area under the curve (AUC) for each mouse’s curve. Energy expenditures were not altered in either sex at room temperature. Interestingly, probing for impact on glucose homeostasis in the gonadal white adipose tissue (gWAT) revealed that *Trsp^f/f^-Ucp1-Cre^+/−^* mice had a reduction in the activation of Akt, as assessed by levels of phosphorylated Akt at serine 473 (pAkt-Ser473) residue (Figure 2g). Reduced activation occurred in male but not female *Trsp^f/f^-Ucp1-Cre^+/−^* mice, even though both sexes had reduced total Akt levels.

**Figure 3:**
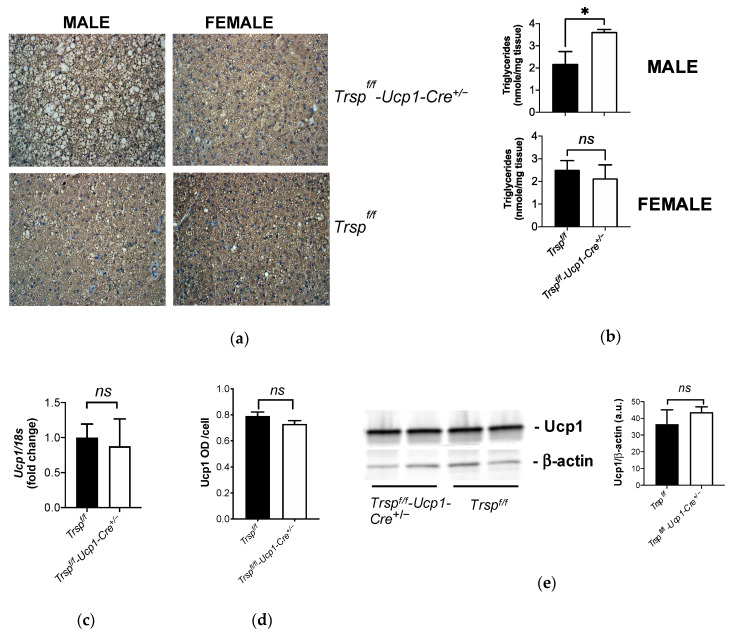
BAT morphology and Ucp1 expression in *Trsp^f/f^-Ucp1-Cre^+/−^* mice. (**a**) Representative images of BAT histology after hematoxylin staining, *n* = 11. (**b**) BAT triglyceride content of *Trsp^f/f^-Ucp1-Cre^+/−^* mice (*n* = 7 per sex). (**c**) *Ucp1* mRNA expression in male *Trsp^f/f^-Ucp1-Cre^+/−^* mice. (**d**) Ucp1 levels as assessed by optical densitometry after Ucp1 immunohistochemistry of male samples in (**a**). (**e**) Western blot (*n* = 11) image of Ucp1 levels and graph with band densitometry calculation. Values are mean ± SD, and *p*-values were calculated using unpaired Student’s *t*-test. *ns*, non-significant; * *p* < 0.05.

Despite unaltered energy expenditure, observation of brown adipocytes from the male *Trsp^f/f^-Ucp1-Cre^+/−^* mice revealed increased lipid deposition compared to either control or female *Trsp^f/f^-Ucp1-Cre^+/−^* mice as indicated by hematoxylin-stained histology (Figure 3a) and measurement of triglyceride content in BAT (Figure 3b). BAT adaptive thermogenesis is conducted by the actions of Ucp1, therefore we examined its gene expression (Figure 3c) and protein levels using both immunohistochemistry (Figure 3d) and Western blotting (Figure 3e). In all analyses, Ucp1 expression and levels were unchanged in *Trsp^f/f^-Ucp1-Cre^+/−^* mice.

### 2.3. Mitochondrial Content in BAT of Trsp^f/f^-Ucp1-Cre^+/−^ Mice

**Figure 4:**
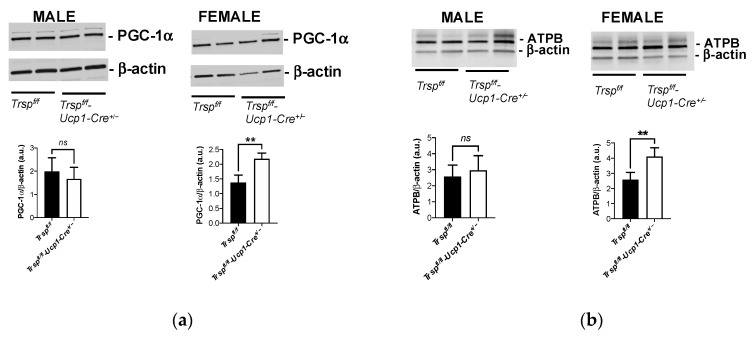
BAT mitochondrial markers in *Trsp^f/f^-Ucp1-Cre^+/−^* mice. Levels of mitochondrial markers (**a**) PGC-1α and (**b**) ATPB in BAT of male and female *Trsp^f/f^-Ucp1-Cre^+/−^* mice were assessed by Western blot (*n* = 8 per sex). Values are mean ± SD and *p*-values were calculated using unpaired Student’s *t*-test. *ns*, non-significant, ** *p* < 0.01.

We next investigated whether unchanged Ucp1 reflected similar mitochondrial content in BAT of *Trsp^f/f^-Ucp1-Cre^+/−^* mice. We employed levels of peroxisome proliferator-activated receptor gamma-coactivator 1-alpha (PGC-1α) and ATP synthase subunit beta (ATPB) as proxies for mitochondrial content and function. PGC-1α is a transcriptional coactivator and central inducer of mitochondrial biogenesis [34], while ATPB is a member of the complex V of the electron transport chain in the inner membrane of the mitochondria and commonly used as a mitochondrial marker. Interestingly, BAT of female *Trsp^f/f^-Ucp1-Cre^+/−^* mice presented increased levels of both PGC-1α (Figure 4a) and ATPB (Figure 4b), while male *Trsp^f/f^-Ucp1-Cre^+/−^* mice kept similar expression levels of both markers.

### 2.4. Changes in Gene Expression and Pathways in BAT of Trsp^f/f^-Ucp1-Cre^+/−^ Mice at Room Temperature

Maintenance of Ucp1 expression in *Trsp^f/f^-Ucp1-Cre^+/−^* mice led us to seek genes and pathways that were either up- or down-regulated in BAT of male *Trsp^f/f^-Ucp1-Cre^+/−^* mice at room temperature. We performed microarray analysis and found 183 genes with at least a 2-fold of change in expression between the two genotypes (Supplementary Table S1). Table 1 shows the top 10 differentially expressed genes in these mice. At room temperature, no genes for selenoproteins were affected by the loss of *Trsp*.

Pathway analysis revealed that, at room temperature, nicotine degradation, antigen presentation, glycerol degradation, LPS/IL-1 mediated inhibition of RXR function, and myo-inositol biosynthesis were the pathways most affected in BAT of *Trsp^f/f^-Ucp1-Cre^+/−^* mice (Table 2 and Supplementary Table S1 for the full list).

### 2.5. Body Temperature in the Trsp^f/f^-Ucp1-Cre^+/−^ Mice after Acute Cold Exposure

**Figure 5:**
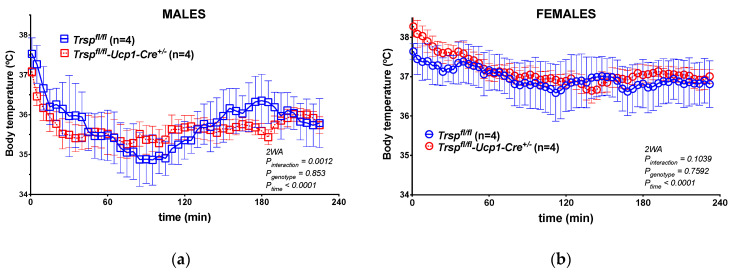
Core body temperature in *Trsp^f/f^* and *Trsp^f/f^-Ucp1-Cre^+/−^* male (**a**) and female (**b**) mice during acute cold exposure. Data are mean ± SD; two-way ANOVA (2WA) with Bonferroni’s post-test was performed, and *p*-values are displayed in graphs, as well as sample sizes.

The phenotype observed in the *Trsp^f/f^-Ucp1-Cre^+/−^* mice at room temperature led us to consider that the effects of *Trsp* loss in brown adipocytes might be revealed to a greater extent after activation of adaptive thermogenesis. Hence, we acutely exposed *Trsp^f/f^-Ucp1-Cre^+/−^* and *Trsp^f/f^* mice to 4 °C for 4 h. Both male and female *Trsp^f/f^-Ucp1-Cre^+/−^* mice reduced their core body temperature at the same rate as corresponding controls (Figure 5). An interaction effect of genotype and time was observed in males (Figure 5a), but not in females (Figure 5b).

### 2.6. Changes in Gene Expression and Pathways in BAT of Trsp^f/f^-Ucp1-Cre^+/−^ Mice after Acute Cold Exposure

Slight differences in core body temperature during acute cold exposure in male *Trsp^f/f^-Ucp1-Cre^+/−^* mice led us to investigate whether male *Trsp^f/f^* and *Trsp^f/f^-Ucp1-Cre^+/−^* mice showed similar changes in gene expression in BAT as when the mice were at room temperature. Interestingly, microarray analyses of BAT from *Trsp^f/f^* and *Trsp^f/f^-Ucp1-Cre^+/−^* mice exposed to cold revealed only 65 differentially expressed genes compared to the 183 differentially expressed genes observed at room temperature (Supplementary Table S2), i.e., one-third of the number of gene expression changes. Table 3 shows the top up- and down-regulated genes in the BAT of *Trsp^f/f^-Ucp1-Cre^+/−^* mice after cold exposure.

**Figure 6:**
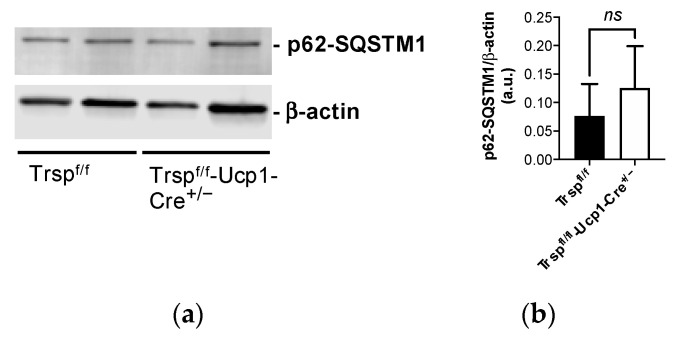
Autophagy in the cold-exposed male *Trsp^f/f^-Ucp1-Cre^+/−^* mice. Levels of autophagy marker p62-SQSTM1 in the BAT of cold-exposed male *Trsp^f/f^-Ucp1-Cre^+/−^* mice were obtained by (**a**) Western blot; (**b**) graph displays quantification analysis. Values are expressed as mean + SD, *n* = 11. Student’s unpaired *t*-test was performed; *ns*, non-significant.

Pathway analysis revealed that the autophagy pathway was the most affected pathway of those examined by cold exposure in the *Trsp^f/f^-Ucp1-Cre^+/−^* mice compared to their controls, followed by histidine degradation III (Table 4 and Supplementary Table S2 for the full list). Nevertheless, levels of scaffold protein p62 (sequestosome 1; p62-SQSTM1), an autophagy marker implicated in the control of BAT thermogenesis [35], remained unchanged in the *Trsp^f/f^-Ucp1-Cre^+/−^* mice, as revealed by Western blot analysis (Figure 6a). Quantitation of the Western blot is shown in Figure 6b.

**Figure 7:**
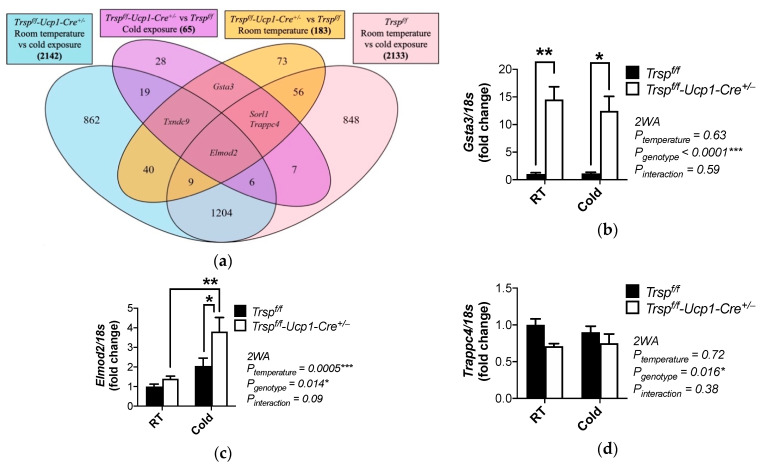
Gene expression changes in *Trsp^f/f^-Ucp1-Cre^+/−^* mice at room temperature and after acute cold exposure. (**a**) Venn diagram of microarray data sets, showing the number of genes differentially expressed between *Trsp^f/f^-Ucp1-Cre^+/−^* and *Trsp^f/f^* mice at room temperature and after cold exposure. (**b**–**d**) Validation of microarray results by qPCR analysis of the expression of *Gsta3*, *Elmod2* and *Trappc4*, respectively. Data are mean ± SD. *p*-values are shown in graphs and were calculated by two-way ANOVA (2WA) followed by Bonferroni’s post test analysis. * *p* < 0.05,** *p* < 0.01, and *** *p* < 0.001. RT, room temperature.

To pinpoint BAT genes that were most heavily affected by the loss of *Trsp* in brown adipocytes, we combined and compared the microarray results from mice maintained at room temperature and mice acutely exposed to cold. Figure 7a shows a Venn diagram built with the differentially expressed genes obtained from the microarray analyses. Our results showed two genes at the main intersection of the diagram, glutathione S-transferase alpha-3 (*Gsta3*) and ELMO domain containing 2 (*Elmod2*). *Gsta3* is a gene regulated by the thyroid hormone in the liver [36] that catalyzes the conjugation of glutathione to several electrophiles, and it was upregulated in the *Trsp^f/f^-Ucp1-Cre^+/−^* mice, regardless of environmental temperature (Figure 7b). On the other hand, *Elmod2*, a GTPase activating protein which regulates adipocyte triglyceride lipase recruitment [37], was found to be significantly upregulated after cold exposure in both genotypes. However, the upregulation occurred with a further ~40% increase in BAT in *Trsp^f/f^-Ucp1-Cre^+/−^* mice (Figure 7c). Loss of *Trsp* also affected the transcription of *Trappc4* (trafficking protein particle complex 4), an interactor of ERK2 implicated in vesicle transport in colorectal carcinoma cells, which was diminished in *Trsp^f/f^-Ucp1-Cre^+/−^* mice (Figure 7d) [38].

### 2.7. Thyroid Function in the Trsp^f/f^-Ucp1-Cre^+/−^ Mice

Activation of adaptive thermogenesis is critically dependent on thyroid hormone availability. We assessed thyroid function in *Trsp^f/f^-Ucp1-Cre^+/−^* mice at room temperature and after acute cold exposure. We found male mice to have unchanged circulating total T4 levels but diminished total T3 levels. Cold exposure reduced total T4 levels but increased total T3 levels in female mice of both genotypes. Interestingly, male *Trsp^f/f^-Ucp1-Cre^+/−^* mice at room temperature had modest elevation of circulating thyroid-stimulating hormone (TSH), a sign of mild hypothyroidism, in comparison to their *Trsp^f/f^* counterparts. Upon acute cold exposure, male mice of both genotypes reduced circulating TSH levels, however a greater reduction was observed in the *Trsp^f/f^-Ucp1-Cre^+/−^* mice (Table 5).

**Figure 8:**
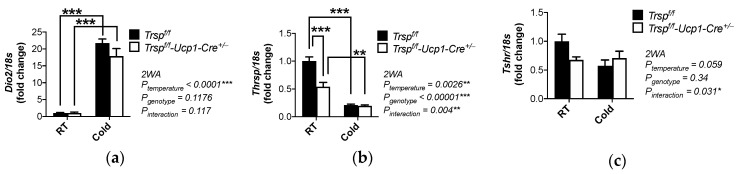
Gene expression of thyroid hormone responsive transcripts in male *Trsp^f/f^* and *Trsp^f/f^-Ucp1-Cre^+/−^* mice**.** Expression levels of (**a**) *Dio2*, (**b**) *Thrsp* (i.e., *Spot14*), and (**c**) *Tshr* were assessed by qPCR. RT, room temperature. Data are mean + SD; *n* = 8–10 per genotype. *p*-values are displayed in graphs and calculated by two-way ANOVA followed by Bonferroni’s post-test. * *p* < 0.05, ** *p* < 0.01 and *** *p* < 0.001.

In BAT, thyroid hormone activation is catalyzed by selenoprotein Dio2, and upregulation of *Dio2* is considered a classic marker of BAT activation. Interestingly, we observed that in BAT of *Trsp^f/f^-Ucp1-Cre^+/−^* mice, which had histological changes suggestive of physiological impairment, *Dio2* expression was higher after cold exposure. However, this increase occurred at the same levels in both genotypes (Figure 8a).

Using our microarray dataset, we next validated by qPCR other thyroid hormone-regulated genes that were differentially expressed in BAT of *Trsp^f/f^-Ucp1-Cre^+/−^* mice. *Thrsp* (thyroid hormone-inducible hepatic protein, also known as Spot14), a classic T3-responsive gene that regulates lipogenesis [39,40] and acts as a transcriptional coactivator with thyroid hormone receptor beta [41], was downregulated in *Trsp^f/f^-Ucp1-Cre^+/−^* mice according to both temperature and genotype (Figure 8b). Moreover, the TSH receptor gene, *Tshr*, also presented a non-significant trend (*p* = 0.057) towards downregulation in the *Trsp^f/f^-Ucp1-Cre^+/−^* mice. However, when acutely exposed to cold, both genotypes showed the same expression levels (Figure 8c).

### 2.8. Methylation Genes in the Trsp^f/f^-Ucp1-Cre^+/−^ Mice

**Figure 9:**
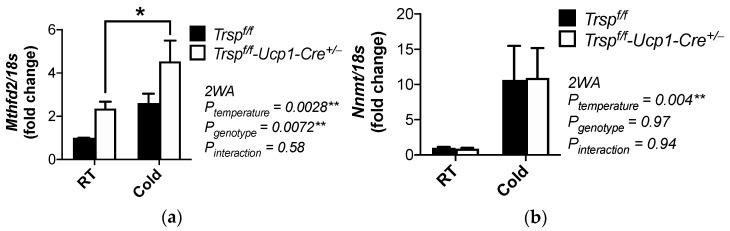
Gene expression of transcripts involved in methylation pathways in male *Trsp^f/f^* and *Trsp^f/f^-Ucp1-Cre^+/−^* mice. Expression of (**a**) *Mthfd2* and (**b**) *Nnmt* were assessed by qPCR. RT, room temperature. Data are mean + SD; *n* = 8–10 per genotype. *p*-values are displayed in graphs and were calculated by two-way ANOVA (2WA) followed by Bonferroni’s post test; * *p* < 0.05, ** *p* < 0.01.

Surprisingly, we found that two genes involved in methylation in cancer cells were altered in our BAT microarray analysis and were selected for validation by qPCR. *Mthfd2*, which encodes for methylenetetrahydrofolate dehydrogenase (NADP+ dependent) 2, participates in one-carbon metabolism. This enzyme also controls RNA methylation, particularly N^6^-methyladenosine (m^6^a) in mRNAs, in renal cell carcinoma [42]. *Mthfd2* expression was upregulated in the *Trsp^f/f^-Ucp1-Cre^+/-^* mice, and further enhanced after acute cold exposure (Figure 9a). On the other hand, *Nnmt,* which encodes nicotinamide N-methyl transferase, is an enzyme that inhibits autophagy in breast cancer cells [43]. It showed a dramatic ~24-fold upregulation in BAT of *Trsp^f/f^-Ucp1-Cre^+/−^* mice after cold exposure compared to corresponding controls (Supplementary Table S2). Our qPCR validation revealed an upregulation dependent solely on cold exposure, without a genotype effect, in BAT of these mice (Figure 9b).

## 3. Discussion

We have reported the development of a novel transgenic mouse model, the *Trsp^f/f^-Ucp1-Cre^+/−^* mice. Our model was characterized by targeted deletion of the gene *Trsp* in brown adipocytes, which are the primary cell type of BAT. The product of the *Trsp* gene, tRNA^[Ser}Sec^, is responsible for carrying out the synthesis of selenoproteins, including Dio2, a regulator of cold-induced adaptive thermogenesis in BAT.

*Dio2* upregulation is promoted by thyroid hormone and is considered a primary indicator of activation of adaptive thermogenesis in cold exposure [44]. In males, *Dio2* expression was unchanged between *Trsp^f/f^* and *Trsp^f/f^-Ucp1-Cre^+/−^* mice, despite a reduction in circulating T3. In addition, circulating T4, the substrate used by Dio2 to generate T3 that, in turn, activates thermogenesis, was unchanged. In BAT, Ucp1 levels were also unchanged. Nevertheless, BAT of male *Trsp^f/f^-Ucp1-Cre^+/−^* mice had a distinct tissue morphology resembling more a white adipocyte with increased lipid accumulation, typical of under-stimulated brown adipocytes [45]. Together, these results suggest that, despite T4 levels not being affected by the loss of *Trsp* in male *Trsp^f/f^-Ucp1-Cre^+/−^* mice, the physiology of their brown adipocytes are indeed impaired. This impairment in the morphology of BAT in males possibly occurs since early development, because mice lacking *Dio2* show permanent defect in adaptive thermogenesis in the embryonic BAT [46].

Murine selenoproteins are expressed in a sexually dimorphic manner in other organs, such as liver and kidneys [47,48]. A possible explanation for the sexual dimorphism we observed in BAT of male *Trsp^f/f^-Ucp1-Cre^+/−^* mice could be alterations in the sympathetic tone in our mouse model. However, one of the regulators of the sympathetic tone of BAT is T4 via central and peripheral mechanisms [49]. However, the *Dio2* knockout mouse model does not present sexual dimorphism in their thermogenic responses after a caloric overload at room temperature [31]. Moreover, T4 is not altered in males, making it unlikely that the sympathetic tone is diminished in BAT of these mice. An alternative to be explored in future studies is whether another selenoprotein, another factor involved in selenium metabolism, or another selenium-related mechanism plays a role in regulating the sympathetic tone in BAT according to sex.

Interestingly, elevated circulating TSH levels in male *Trsp^f/f^-Ucp1-Cre^+/−^* mice at room temperature suggest either impaired thyroid stimulation to produce T4—which does not occur as T4 levels are maintained—or a direct effect of TSH via its receptor TSHR in brown adipocytes, which could explain the morphological changes of BAT. Such direct effect of TSH has been demonstrated in rats to positively modulate oxygen consumption and inversely regulate *Dio2* mRNA [50]. However, this observation is discrepant with our results herein in the male *Trsp^f/f^-Ucp1-Cre^+/−^* mice, as circulating TSH were higher but effects on neither oxygen consumption nor *Dio2* mRNA were observed. It is possible that other mechanisms modulated by TSH alone, such as activation of Erk or Akt phosphorylation, are recruited [50], reducing the expected responses cited above.

Acute cold exposure leads to intense lipolysis, adipocyte autophagy, and remodeling of BAT, using the lipid deposits available to increase the mitochondrial oxidative cycle. This, in turn, could produce heat, maintaining the animal’s core body temperature and avoiding hypothermia. Extensive use of lipid deposits leads to activation of a lipogenic phase with enhanced lipid uptake in brown adipocytes, guaranteeing fuel for thermogenesis maintenance as cold exposure continues [51]. Despite BAT undergoing intense remodeling in the first hours of cold exposure, it is intriguing that male *Trsp^f/f^-Ucp1-Cre^+/−^* mice do not present alterations in the autophagy flux; levels of autophagy factor p62/SQSTM1 were sustained in male *Trsp^f/f^-Ucp1-Cre^+/−^* mice, while autophagy genes were upregulated in our microarray analysis. This discrepancy suggests either a reduction in translational efficiency of autophagic transcripts or a temporal dissonance in autophagy activation in male *Trsp^f/f^-Ucp1-Cre^+/−^* mice acutely exposed to cold. The varying length of cold exposure has been considered as a source of conflicting data for autophagy response in adaptive thermogenesis ([52] citing [53,54,55]). These studies did not expose mice for 4 h in the cold; therefore, it is also possible that we uncovered a transitional moment for autophagy. However, male *Trsp^f/f^-Ucp1-Cre^+/−^* mice accumulated more triglycerides than control mice, despite similar levels of mitochondrial markers at room temperature. These combined findings suggest that male *Trsp^f/f^-Ucp1-Cre^+/−^* mice maintained mitochondrial capacity with excess resources for the initial lipolytic phase of adaptive thermogenesis. It is possible that during cold exposure, as brown adipocytes switch from lipolysis to lipogenesis, the advantage provided by additional fuel is counteracted by deficiencies in thermogenic responses that are dependent on other factors, such as thyroid hormone activation or sympathetic tone [56].

It is puzzling that the loss of *Trsp*, i.e., selenoprotein synthesis capacity, does not lead to a more dramatic impact in gene expression, particularly of selenoproteins after acute cold exposure. As mentioned in the previous paragraph, the late lipogenic phase of chronic cold exposure depends on the proper transcriptional activation of T3-responsive genes encoding key proteins involved in lipogenesis early on, which will later allow for withstanding the constant temperature stress. Our finding that the *Thrsp* transcript, which encodes a coactivator of lipogenic genes via interaction with the thyroid hormone receptor [40,41], was downregulated in *Trsp^f/f^-Ucp1-Cre^+/−^* mice suggests lower availability of thyroid hormones locally in the early stages of cold exposure. It is plausible to infer that these mice, upon chronic exposure to cold, may diminish their thermogenic capacity due to impaired lipid uptake, because the proteins required for lipogenesis could be at lower levels. Prospective studies examining the chronic activation of BAT adaptive thermogenesis, either in a more physiological manner after chronic cold exposure or in a pathological context, after a high-fat diet, may consolidate whether observed changes in thyroid hormone response of the *Trsp^f/f^-Ucp1-Cre^+/−^* mice are sustained. Moreover, the participation of additional gut hormones in concert with the sympathetic response may mediate chronic thermogenic responses. A chronic paradigm for adaptive thermogenesis activation, particularly in a pathological context after a hypercaloric overload, may allow for display of a worsened metabolic dysfunction. We have observed a moderate impairment of Akt activation, i.e., in insulin response, in the gWAT of *Trsp^f/f^-Ucp1-Cre^+/−^* mice. Therefore, it is likely that the feeding of a high-fat diet will accelerate the development of a metabolic dysfunction in a *Trsp^f/f^-Ucp1-Cre^+/−^* mouse.

An interesting aspect found by our microarray analysis was the upregulation of genes involved in methylation. As occurs with other tRNAs [57], a portion of the tRNA^[Ser]Sec^ population is methylated at the uridine position 34 (Um34) yielding a specific subpopulation [58]. In fact, differential methylation of Um34 leads to the existence of two possible molecular conformations in relation to the anticodon sequence [59]. Consequently, two subsets of selenoproteins, deemed stress-related and housekeeping, occur, with tRNA^[Ser]Sec^ with Um34 supporting primarily translation of stress-related selenoproteins. Members of this class of selenoproteins are sensitive to selenium status [20,21], which is likely the case for Dio2 in the activated BAT. The specific methyltransferase responsible for the methylation of Um34 has not been identified, although one that is sensitive to S-adenosyl-homocysteine accumulation has been indicated as a promising candidate [60]. In our mouse model, loss of tRNA^[Ser]Sec^ in brown adipocytes led to upregulation of *Mthfd2*, which encodes an enzyme from folate one-carbon metabolism. This enzyme also binds to several ribonucleoproteins and is involved in protein translation [61], controlling the methylation of N^6^-methyladenosine (m^6^a) of mRNAs [42]. It is tempting, at this point, to suggest that this enzyme may be an additional promising candidate for at least controlling the Um34 methylation of tRNA^[Ser]Sec^.

Considering the impairment of selenoprotein synthesis in the brown adipocyte, it is intriguing that a general decline in selenoprotein gene expression, particularly *Dio2*, is not observed. Stress-related selenoprotein mRNAs, such as *Dio2*, are primary targets for nonsense-mediated decay (NMD) degradation, a mechanism that misreads the in-frame UGA codon of selenoproteins as a nonsense codon [62]. Selenium deficiency further activates the NMD machinery to target selenoprotein mRNAs for degradation [63]. *Trsp^f/f^-Ucp1-Cre^+/−^* mice were fed diets with adequate selenium, which likely provided enough selenium to inhibit NMD machinery, allowing selenoprotein mRNAs to circumvent NMD degradation.

Moderate dietary selenium intake in *Trsp^f/f^-Ucp1-Cre^+/−^* mice also raises a striking possibility regarding the fate of selenium in brown adipocytes. With the impairment of selenoprotein synthesis, available selenium may be either released back into circulation or to neighboring cell types, or redirected within the brown adipocyte for use in additional pathways. Jedrychowski et al. [32] have demonstrated that excess selenium in brown adipocytes impacts adaptive thermogenesis via incorporation of Sec in the place of cysteine in several non-selenoproteins, i.e., facultative selenation. Notably, cysteine residue 253 of Ucp1 was identified to be selenated. Selenation of Ucp1 increases its redox sensitivity, enhancing energy expenditure, activating adaptive thermogenic responses, and protecting from high-fat diet-induced obesity. It is possible that selenium not used in selenoprotein synthesis by the *Trsp^f/f^-Ucp1-Cre^+/−^* mice enhances facultative selenation. Enhanced facultative selenation, particularly of Ucp1, could explain the mild effects of acute cold exposure observed in our mouse model, with the lack of *Trsp* and consequent selenoprotein synthesis freeing selenium to be redirected towards selenation in brown adipocytes. It is unknown whether facultative selenation in brown adipocytes requires tRNA^[Ser]Sec^. We believe that the *Trsp^f/f^-Ucp1-Cre^+/−^* mouse model may be fitting to test this hypothesis in the future.

A limitation of this study encompasses the inability to breed or maintain mice at their thermoneutrality (~28–30 °C) due to the lack of this capacity at our animal facilities. Mice maintenance at temperatures slightly lower than thermoneutrality could have primed their BAT to activate thermogenic mechanisms. Such prior activation could diminish the impact of acute cold exposure, and this effect was observed in a mouse model lacking *Dio2* [64]. Despite this limitation, we still observed subtle changes at the transcriptional level after acute cold exposure, particularly in genes responding to thyroid hormone and involved in methylation pathways. The fact that we unveiled changes in gene expression between the *Trsp^f/f^-Ucp1-Cre^+/−^* and *Trsp^f/f^* control mice indicates that the effects of this temperature priming were potentially minimized.

In summary, we developed a novel mouse model lacking the *Trsp* gene in brown adipocytes and demonstrated that loss of *Trsp* in these cells leads to a modest impairment of acute cold exposure responses in male *Trsp^f/f^-Ucp1-Cre^+/−^* mice, but not in females. We also observed subtle changes in thyroid hormone responsiveness and genes involved in methylation pathways. Future studies moving towards a chronic cold exposure may help refine our knowledge of the role of selenoproteins in rodent adaptive thermogenesis.

## 4. Materials and Methods

### 4.1. Chemicals and Antibodies

All chemicals used in experiments were from Fisher Scientific (Hampton, NH, USA) or Sigma-Aldrich (St. Louis, MO, USA), unless specified. Primary antibodies included anti-β-actin (1:3000; cat. #A2228; Sigma-Aldrich), anti-Ucp1 (1:1000; cat. #ab10983, Abcam; Cambridge, MA, USA), anti-PGC1α (1:750; cat. #AB3242, MilliporeSigma; Burlington, MA, USA), anti-ATPB (1:1000; cat. #ab14730; Abcam), anti-Akt (1:1000; cat. #9272, Cell Signaling Technology, Beverly, MA, USA); anti-phospho-Akt-Ser473 (1:750; cat#9271, Cell Signaling Technology), and anti-p62/SQSTM1 (1:750; cat. #5114T, Cell Signaling Technology).

### 4.2. Animals

Animal procedures were approved by the University of Hawaii Office of Research Compliance Animal Welfare Program, protocol #17-2521, first approved by the Institutional Animal Care and Use Committee on 19 October 2017. Mice were held in our vivarium and used for experiments in minimal numbers needed to provide significant results. B6.FVB-Tg(Ucp1-cre)1Evdr/J (MGI J:206508; shortened here as *Ucp1*-*Cre*) mice on a C57BL6/J background were purchased from The Jackson Laboratory (cat. #024670; Bar Harbor, ME, USA). *Trsp*-*LoxP* were generated as previously described [65]. To generate a strain with a targeted deletion of *Trsp* in brown adipocytes, the *Trsp^f/f^-Ucp1-Cre^+/−^* mice, *Trsp^f/+^* mice were crossed with *Ucp1*-*Cre* mice. Homozygote *Trsp^f/f^* littermate controls were used to compare with *Trsp^f/f^-Ucp1-Cre^+/−^* mice after genotyping confirmation by PCR. Mice were in a 12 h light–dark cycle, fed *ad libitum* with chow containing ~0.25 ppm sodium selenite, and group-caged until exposure to cold. Euthanasia occurred at 12-weeks old by CO_2_ asphyxiation, and all mice were euthanized ~2 pm, to avoid circadian influences in results. After euthanasia, blood and interscapular BAT, which comprises approximately 60% of all BAT depots and considered the most significant in mice [66], were removed immediately, snap-frozen into liquid nitrogen, perfused with formalin, or incubated in RNALater Stabilization Solution (Thermo Fisher Scientific; Waltham, MA, USA).

### 4.3. Energy Expenditure Assessment

Oxygen consumption (VO_2_), respiratory quotient (RQ) and energy expenditure (EE) were assessed for 24 h with the PanLab OxyLet*Pro^TM^* System (Harvard Apparatus, Barcelona, Spain) in 11-week-old mice. Mice were habituated in individual cages for 24 h prior to running experiments, as previously described [67]. Data were analyzed using PanLab Metabolism (Prague, Czech Republic) software.

### 4.4. Cold Exposure and Core Body Temperature Assessment

A G2 emitter thermal probe (Starr Life Sciences Corp.; Oakmont, PA, USA) was surgically inserted into the peritoneal cavity of mice one week prior to exposure to cold. Only mice that adequately recovered from survival surgery were used in experiments. On the day of experiment, 12-week-old mice were transferred to individual cages with only water and minimal bedding, transported to a cold room set at 4 °C between 09:00 and 10:00 a.m., and each cage placed on a receiver telemetry platform (Starr Life Sciences Corp.). Mice remained in the cold room uninterrupted for 4 h and were euthanized ~2 pm. Core body temperature was recorded every 5 min by the receiver using Vital View Legacy Version 5.1 software (Starr Life Sciences Corp.).

### 4.5. Triglyceride Content

Triglyceride content of BAT was measured using the Triglyceride Quantification Assay Kit (Abcam) for colorimetric detection, following the manufacturer’s protocol.

### 4.6. RNA Extraction

Mouse interscapular BAT was collected at time of euthanasia (~2pm) either in RNALater (Thermo Fisher Scientific) or snap-frozen, according to final use. For quantitative PCR (qPCR), total RNA was extracted from snap-frozen tissues using a TissueRuptor (Qiagen; Germantown, MD, USA) with disposable probes, and isolated using the EZNA total RNA kit I (Omega Biotek; Norcross, GA, USA). For microarray, total RNA was extracted from tissues in RNALater, processed with TissueRuptor and isolated using the Qiagen RNA extraction kit (Qiagen).

### 4.7. Microarray Analysis

RNA quality was assessed in an Agilent 2100 BioAnalyzer (Agilent Technologies; Santa Clara, CA, USA) and samples with RIN > 5.5 were deemed satisfactory for microarray. Mouse ClariomS Array gene hybridization (Affymetrix—Thermo Fisher Scientific) was carried out with 100 ng of total RNA, run and scanned in GeneChip Fluidics Station 450 and a GeneChip Scanner (Affymetrix—Thermo Fisher Scientific) in the Genomics and Bioinformatics Shared Resource facility at the University of Hawaii Cancer Center. The CEL files were processed and analyzed using the Transcriptome Analysis Console (TAC) 4.0 from Thermo Fisher Scientific. Genes with a fold change greater than 2 and *p*-value less than 0.05 were considered differentially expressed. Canonical pathway and network analysis of differentially expressed genes was performed using Qiagen’s Ingenuity Pathway Analysis (IPA; Qiagen; https://www.qiagenbioinformatics.com/products/ingenuity-pathway-analysis).

### 4.8. Real-Time qPCR

One microgram of total RNA extracted from the interscapular BAT was reverse transcribed using the High Capacity kit (Applied Biosystems—Thermo Fisher Scientific). 10 ng of cDNA were used in real-time qPCR reactions with the PerfeCTa SYBR Green SuperMix (Quantabio; Beverly, MA) and specific primers, listed in Supplementary Table S4. Relative quantification of target gene expression was calculated based on the Δ^Ct^ method, normalized to the expression of housekeeping genes for *18s* or *Gapdh*, and plotted as fold change relative to *Trsp^f/f^* at room temperature values.

### 4.9. Immunohistochemistry

Mice acclimated to room temperature had their BAT extracted after perfusion with formalin and were paraffin-embedded for histological analysis. Immunohistochemistry was performed in slides using citrate buffer in a pressure cooker. Slides were labeled with primary antibody (anti-Ucp1; 1:100), Mouse on Mouse Basic kits with Vectastain ABC, and diaminobenzidine peroxidase substrate kits (Vector Labs; Burlingame, CA, USA) as previously described [68]. Slides were counter-stained with hematoxylin to visualize adipocyte nuclei.

Four brightfield images were acquired from a single section of BAT from each subject at 40× magnification using the simple random sampling workflow in Stereo Investigator (MBF Bioscience; Williston, VT, USA). To compare Ucp1 immunoreactivity, the optical density of each image was quantified using ImageJ (public domain) and averaged for each subject. To calculate Ucp1 optical density, images were converted to black-and-white, inverted, and the mean pixel value for the entire image recorded. Total nuclei were counted using ImageJ Cell Counter plugin (public domain) and used to normalize the mean pixel value for each image.

### 4.10. Western Blot

BAT and gWAT protein were extracted using RIPA Lysis and Extraction buffer containing protease and phosphatase inhibitors (Thermo Scientific) followed by sonication and two rounds of centrifugation. 10–20 μg of total protein was loaded into 4–20% TGX SDS-PAGE (Bio-Rad; Hercules, CA, USA) and wet-transferred overnight in Tris-glycine buffer containing 9% methanol to an Immobilon-FL^®^ membrane (MilliporeSigma) using the Criterion Blotter (Bio-Rad) apparatus. Primary and secondary antibodies were incubated for 1 h at room temperature with rotation, and secondary antibodies were IRDyes (Li-Cor Biosciences; Lincoln, NE) for use at an Odyssey CTx infra-red scanner (Li-Cor Biosciences). All blots were used only once, except the measurement of phosphorylated Akt, performed on the same membrane after confirmation of complete stripping of previous antibodies using NewBlot PVDF Stripping Buffer (Li-Cor Biosciences). Analysis of the Western blot was conducted with the Image Studio Lite version 5.2.5. software (Li-Cor Biosciences).

### 4.11. Thyroid Function

Thyroid function was inferred from analyses of total T4, total T3, and TSH levels in the serum. Twenty microliters of serum were used to assay for TSH with a Milliplex MAP Mouse Pituitary Magnetic Bead Panel (Millipore-Sigma) on a Luminex 200 platform (Luminex; Austin, TX, USA). Twenty-five microliters of serum were used to assay for total T4 and total T3 using the AccuDiag^TM^ ELISA—T4 kit (Diagnostic Automation; Woodland Hills, CA, USA) and the T3 (Total) ELISA kit (Abnova; Taipei, Taiwan), respectively, according to the manufacturer’s instructions.

### 4.12. Statistical Analysis

Results were plotted and graphed using Graphpad Prism v. 8.0 (San Diego, CA), and an alpha value of 0.05 was adopted. Unpaired Student’s *t*-test or two-way analysis of variance (2WA) with Bonferroni’s *ad hoc* post-test were used according to the number of variables analyzed in each experiment.

## Figures and Tables

**Table 1:** Top differentially expressed genes in BAT of *Trsp^f/f^-Ucp1-Cre^+/−^* mice at room temperature.

Top Upregulated			
**Gene Symbol**	**Gene Name**	**Fold Change**	***p*-Value**
*Plekhm2*	pleckstrin homology and RUN domain containing M2	4.59	0.0361
*Sntb2*	syntrophin beta 2	4.02	0.0008
*Phf20*	PHD finger protein 20	3.78	0.0271
*Vps4a*	vacuolar protein sorting 4 homolog A	3.33	0.0296
*Fads3*	fatty acid desaturase 3	3.3	0.0119
*S100pbp*	S100P binding protein	3.26	0.0005
*Lcp1*	lymphocyte cytosolic protein 1	3.17	0.0453
*Fxyd5*	FXYD domain containing ion transport regulator 5	3.07	0.0334
*Elmod2*	ELMO domain containing 2	2.79	0.003
*Kpna2*	karyopherin subunit alpha 2	2.72	0.0061
**Top Downregulated**			
**Gene Symbol**	**Gene Name**	**Fold Change**	***p*-Value**
*Tmsb4x*	thymosin, beta 4, X chromosome	−4.24	0.0172
*Trappc4*	trafficking protein particle complex 4	−3.63	0.0183
*Hscb*	HscB mitochondrial iron-sulfur cluster cochaperone	−3.12	0.0196
*Ccdc85a*	coiled-coil domain containing 85A	−2.98	0.003
*Skil*	SKI-like proto-oncogene	−2.87	0.0297
*Sorl1*	sortilin related receptor 1	−2.8	0.0299
*Gk*	glycerol kinase	−2.66	0.0094
*Nbea*	neurobeachin	−2.46	0.0228
*Tmx4*	thioredoxin related transmembrane protein 4	−2.43	0.0234
*Per2*	period circadian regulator 2	−2.42	0.0481

**Table 2:** Top 5 pathways affected in BAT of *Trsp^f/f^-Ucp1-Cre^+/−^* mice at room temperature.

Canonical Pathways	−log(*p*-Value)	Molecules
Nicotine Degradation II	1.85	FMO2,FMO5
Antigen Presentation Pathway	1.78	HLA-DMB,MR1
Glycerol Degradation I	1.59	Gk
LPS/IL-1 Mediated Inhibition of RXR Function	1.58	GSTA3,FMO2,SCARB1,FMO5
Myo-inositol Biosynthesis	1.47	IMPAD1

**Table 3:** Top differentially expressed genes in BAT of *Trsp^f/f^-Ucp1-Cre^+/−^* mice acutely exposed to cold.

Top Upregulated				
**Gene Symbol**	**Gene Name**	**Fold Change**		***p*-Value**
*Nnmt*	nicotinamide N-methyltransferase	24.07		0.0012
*Elmod2*	ELMO domain containing 2	4.1		0.0018
*Gsta3*	glutathione S-transferase alpha 3	4.05		0.0038
*Laptm5*	lysosomal protein transmembrane 5	3.15		0.0029
*Lyz*	lysozyme	3.15		0.0087
*Ear2*	eosinophil-associated, ribonuclease A family, member 2	2.74		0.0013
*Mthfd2*	methylenetetrahydrofolate dehydrogenase (NADP+ dependent) 2	2.69		0.0126
*Rab3a*	RAB3A, member RAS oncogene family	2.6		0.0131
*Sorl1*	sortilin related receptor 1	2.44		0.0467
*Ctss*	cathepsin S	2.41		0.0376
**Top Downregulated**				
**Gene Symbol**	**Gene Name**	**Fold Change**	***p*-Value**	
*Mbp*	myelin basic protein	−3.17	0.0408	
*Gucd1*	guanylyl cyclase domain containing 1	−2.86	0.0375	
*Rnf149*	ring finger protein 149	−2.56	0.0272	
*Rnf125*	ring finger protein 125	−2.54	0.0276	
*Thrsp*	thyroid hormone responsive (Spot14)	−2.47	0.0044	
*Tshr*	thyroid stimulating hormone receptor	−2.46	0.0266	
*Insig1*	insulin induced gene 1	−2.43	0.0413	
*Art3*	ADP-ribosyltransferase 3	−2.39	0.0255	
*Il18*	interleukin 18	−2.36	0.0035	
*Akap12*	A-kinase anchoring protein 12	−2.35	0.0085	

**Table 4:** Top 5 pathways affected in BAT of *Trsp^f/f^-Ucp1-Cre^+/−^* mice after acute cold exposure.

Canonical Pathways	−log(*p*-Value)	Molecules
autophagy	2.13	WDFY3,CTSS
Histidine Degradation III	1.94	MTHFD2
Tetrahydrofolate Salvage from 5,10-methenyltetrahydrofolate	1.81	MTHFD2
Folate Transformations I	1.72	MTHFD2
LXR/RXR Activation	1.58	IL18,LYZ

**Table 5:** Thyroid function of the *Trsp^f/f^-Ucp1-Cre^+/-^* mice.

	Room Temperature		Cold Exposure		Two-Way ANOVA		
	*Trsp^f/f^*	*Trsp^f/f^-Ucp1-Cre^+/−^*	*Trsp^f/f^*	*Trsp^f/f^-Ucp1-Cre^+/−^*	P_g_	P_t_	P_i_
TSH (pg/mL)							
Males	1895.2 ± 86.0	2036 ± 46.05 ^a^	1312 ± 112.9	1161 ± 211.7 ^b^	0.861	<0.0001	0.031
Females	710.3 ± 75.4	541.1 ± 282.8	363.7 ± 107.2	503.8 ± 149.4	0.043	0.868	0.094
T4 (μg/dl)							
Males	3.768 ± 1.18	4.06 ± 1.01	3.447 ± 0.46	3.16 ± 0.61	0.994	0.154	0.487
Females	3.661 ± 0.65	3.421 ± 0.42	3.082 ± 0.58	3.009 ± 0.19	0.488	0.038	0.712
T3 (ng/mL)							
Males	1.02 ±0.19	0.79 ± 0.34	1.08 ± 0.24	0.87 ± 0.21	0.01	0.42	0.9
Females	0.62 ± 0.24	0.58 ± 0.1	0.86 ± 0.22	0.75 ± 0.04	0.36	0.01	0.62

Results are expressed as mean ± SD, *n* = 9–10 per genotype. Bold indicates significant *p*-values calculated by two-way ANOVA. ^a^ *p* < 0.05 *Trsp^f/f^* vs. *Trsp^f/f^-Ucp1-Cre^+/−^* mice at room temperature; and ^b^ *p*< 0.05 room temperature vs. cold exposure for *Trsp^f/f^-Ucp1-Cre^+/−^* mice, after Bonferroni’s post-test. *Pg*, *p*-value for the genotype factor; *Pt*, *p*-value for the temperature factor; *Pi*, *p*-value for interaction of both factors; TSH, thyroid-stimulating hormone.

## Data Availability

The data presented in this study are available upon request to the corresponding author.

## Supplementary Materials

The following are available online at https://www.mdpi.com/1422-0067/22/2/611/s1, Table S1: Differentially expressed genes, networks, and pathways in BAT of *Trspf/f-Ucp1-Cre+/−* mice at room temperature obtained by microarray analysis. Supplementary Table S2: Differentially expressed genes, networks, and pathways in BAT of *Trspf/f-Ucp1-Cre+/−* mice after acute cold exposure obtained by microarray analysis. Supplementary Table S3: All comparisons between differentially expressed genes of microarray analysis. Supplementary Table S4: Mouse primer sequences used in this study. Original gel images are also uploaded within Supplementary Materials.

## Abbreviations

BAT	Brown adipose tissue
Dio2	Iodothyronine deiodinase type 2
qPCR	Quantitative PCR
Sec	Selenocysteine
T3	3,3′,5-triiodothyronine
T4	Thyroxine
TSH	Thyroid-stimulating hormone
Ucp1	Uncoupling protein 1
