# Supplementary material for: Adaptive Thermogenesis in a Mouse Model Lacking Selenoprotein Biosynthesis in Brown Adipocytes
Source: Int J Mol Sci. 2021 Jan 9;22(2):611. doi: 10.3390/ijms22020611 (PMC7827413; doi:10.3390/ijms22020611)
Supplement: Supplementary file 1 [file ijms-22-00611-s001.zip › ijms-1012054-SI.pdf]

Supplementary Table D. Mouse primer sequences used in this study.

Gene	Forward	Reverse
18s	CGATTGGATGGTTTAGTGAGG	AGTTCGACCGTCTTCTCAGC
Dio2	CAGTGTGGTGCACGTCTCCAATC	TGAACCAAAGTTGACCACCAG
Elmod2	CGAGAAGGACACAAGTTTTC	AGAGTCATAGGGCTTTTTCC
Gsta3	CCTTTGAAAAGGTGTTGAAG	TCCACATGGTAGAGGAGTTC
Gapdh	TGACATCAAGAAGGTGGTGAA	CCCTGTTGCTGTAGCCGTATTC
Mthfd2	AATTCAATCAGGAAGCTGAA	ATCAACATCCTTGTCAGGAG
Nnmt	CTTCTCTCTGCCTGTGAGTC	TCAACTTCTCCTCCTTCTCA
Thrsp (Spot14)	AAGGATCAGTAGGAAGCACA	TAGATACAGCATCCCTGCTT
Trappc4	CTGACAGGGATCAAGTTTGT	GAGCTAGCTTCAGGTTTTGA
Trsp	GGATGATCCTCAGTGGTCTGGG	GAACCACTCTGTCGCTAAACAGCTAC
Trsp (genotyping)	GCAACGGCAGGTGTCGCTCTGCG	CGTGCTCTCTCCACTGGCTCA
Tshr	TCTGAAGACCATACCCAGTC	TCCGGATTTCTATGTGAGTC
Ucp1	ACTGCCACACCTCCAGTCATT	CTTTGCCTCACTCAGGATTGG
